# To infinity and beyond: Strategies for fabricating medicines in outer space

**DOI:** 10.1016/j.ijpx.2022.100121

**Published:** 2022-06-16

**Authors:** Iria Seoane-Viaño, Jun Jie Ong, Abdul W. Basit, Alvaro Goyanes

**Affiliations:** a Department of Pharmaceutics, UCL School of Pharmacy, University College London, 29-39 Brunswick Square, London WC1N 1AX, UK; b Department of Pharmacology, Pharmacy and Pharmaceutical Technology, Paraquasil Group (GI-2109), Faculty of Pharmacy, Health Research Institute of Santiago de Compostela (IDIS), University of Santiago de Compostela (USC), Santiago de Compostela 15782, Spain; c FabRx Ltd., 3 Romney Road, Ashford, Kent TN24 0RW, UK; d Departamento de Farmacología, Farmacia y Tecnología Farmacéutica, I+D Farma Group (GI-1645), Facultad de Farmacia, The Institute of Materials (iMATUS) and Health Research Institute of Santiago de Compostela (IDIS), Universidade de Santiago de Compostela (USC), Santiago de Compostela, 15782, Spain.

**Keywords:** Future fabrication of pharmaceuticals, Additive manufacturing of drug products, Advanced drug delivery systems and technologies, Pharmacy in space, Extra-terrestrial design of formulations, Stability of medicines, Clinical pharmacokinetics and pharmacodynamics

## Abstract

Recent advancements in next generation spacecrafts have reignited public excitement over life beyond Earth. However, to safeguard the health and safety of humans in the hostile environment of space, innovation in pharmaceutical manufacturing and drug delivery deserves urgent attention. In this review/commentary, the current state of medicines provision in space is explored, accompanied by a forward look on the future of pharmaceutical manufacturing in outer space. The hazards associated with spaceflight, and their corresponding medical problems, are first briefly discussed. Subsequently, the infeasibility of present-day medicines provision systems for supporting deep space exploration is examined. The existing knowledge gaps on the altered clinical effects of medicines in space are evaluated, and suggestions are provided on how clinical trials in space might be conducted. An envisioned model of on-site production and delivery of medicines in space is proposed, referencing emerging technologies (e.g. Chemputing, synthetic biology, and 3D printing) being developed on Earth that may be adapted for extra-terrestrial use. This review concludes with a critical analysis on the regulatory considerations necessary to facilitate the adoption of these technologies and proposes a framework by which these may be enforced. In doing so, this commentary aims to instigate discussions on the pharmaceutical needs of deep space exploration, and strategies on how these may be met.

## Highlights

• Space is a hostile environment that threatens human health and drug stability.
• Data on the behaviour of medicines in space is critical but lacking.
• Novel drug manufacturing and delivery strategies are needed to safeguard crewmembers’ safety.
• Chemputing, synthetic biology, and 3D printing are examples of such emerging technologies.
• A regulatory framework for space medicines must be implemented to assure quality.

## Graphical abstract

**Figure:**
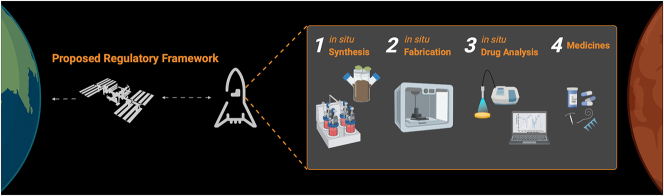

## 1 Introduction

Over 50 years ago, the Apollo 11 Moon Landing inspired millions to imagine life beyond Earth. However, in contrast to the rapid pace at which the Apollo Project saw success, space exploration has remained largely stagnant for the past five decades. This is despite the entry of new government space agencies, such as the European Space Agency (ESA) and the Japan Aerospace Exploration Agency (JAXA), on top of the two key players of the 20^th^-century Space Race, namely the (US) National Aeronautics and Space Administration (NASA) and the Roscosmos (then the Soviet space program). Activities by major government space agencies have largely focused on unmanned explorations such as NASA’s Mars Exploration Program, or crewed space flights to the International Space Station (ISS). Nonetheless, recent developments in next generation spacecrafts and reusable launch systems by private aerospace companies, such as *SpaceX*, have spurred a new era of deep space exploration, with the ambition of launching crewed space flights to Mars in the next 5–10 years (Do et al., 2016; SpaceX, 2021).

Private space companies are leading the development of the next generation of spacecrafts, with applications ranging from deep space exploration (e.g. *SpaceX*) to affordable satellite deployments (e.g. *Rocketlab*). Significant strides have been made towards the goal of landing the first human on Mars, reigniting public excitement towards space exploration. In 2020, *SpaceX* launched the first NASA crew rotation flight to the ISS on an American commercial spacecraft (the *SpaceX* Crew Dragon spacecraft), ending almost a decade of American dependence on the Russian Soyuz spacecraft for such missions (NASA, 2020b). The success of the Crew Dragon also marked the first, and to-date only, reusable crewed or cargo spacecraft. It also set precedence in 2021 for private space tourism through the Inspiration4 mission, which saw four private citizens on board a successful orbital spaceflight. For deep space exploration, *SpaceX* is developing and testing a fully reusable transportation system known as Starship for both crew and cargo, with the aim of Mars colonization (Denis et al., 2020).

While significant progress in aerospace engineering is being made to realize deep space exploration and space tourism, more attention needs to be paid towards the unique medical problems faced during space travel. Astronauts experience prolonged exposure to space radiation, microgravity, isolation, and confinement (Afshinnekoo et al., 2020). These hazards and stresses cause unwanted systemic and physiological effects, such as increased cancer risk, muscle degeneration and bone loss, cardiovascular and circadian rhythm dysregulations, and central nervous system impairments. Critically, in a medical emergency, an urgent return to Earth is impossible (Grigoriev et al., 1998; Houtchens, 1993). Despite the strict medical and psychological standards that astronaut candidates must meet prior to selection, several ailments are still consistently encountered, highlighting the need for medication on board. However, these physiological changes could in return lead to alterations in pharmacokinetic and/or pharmacodynamic profiles of drugs administered in space. As such, alterations to drug doses and release profiles might be necessary to achieve desirable clinical efficacy. Yet, despite more than five decades of manned spaceflights, few data are available on drug pharmacokinetics in space (Kast et al., 2017).

The extended period away from Earth during deep space exploration missions also warrants a thorough evaluation of the long-term storage of medicines, and specially, their on-site production. Medical supplies in Space are currently maintained through regular resupply missions to the ISS, but this system cannot support missions to Mars (and beyond) given the significantly longer distance. Medicines also typically have shelf lives that range between 1 and 5 years (Lyon et al., 2006), which would be inadequate for long-term missions especially if a temporary residence on Mars is planned. Another concern is how prolonged exposure to space radiation could affect drug stability, alter components, or even create toxic by-products. Simple strategies such as using adequate packaging, storing excipients and drugs separately and in their solid or powdered form, or storing them at cryogenic temperatures, could help maintain drug stability. On-site production of medicines could also address these concerns, but to do so, the infrastructure and systems by which medicines are manufactured must be adapted to work in zero and varying magnitudes of gravity.

As humanity looks to Mars and beyond, technologies that are out-of-this-world are being explored as potential solutions to the challenges of deep space exploration. Herein, an overview of the hazards and medical problems associated with spaceflight that warrant medical intervention. We subsequently address how the pharmacokinetic and pharmacodynamic changes that can occur in space due to the previously described physiological changes. Present-day strategies for the manufacture, storage, and management of medicines in space are introduced to set the scene, before providing a forward look on emerging technologies that could be adopted to support on-demand manufacturing of medicines and medical devices. These technologies are namely Chemputing, synthetic biology, and 3D printing. Finally, we leave readers with our perspectives on the prospects of medicine manufacturing in space, including a discussion on the regulatory hurdles that need to be tackled for successful implementation.

## 2 Spaceflight hazards and medical problems

Much of our understanding of what happens to the human body in space is derived from research conducted by NASA’s Human Research Program, in partnership with the Translational Research Institute for Space Health (TRISH) (NASA, 2019d). Prominent studies by NASA’s Human Research Program include the Scott Kelly One-Year Mission, which was the first study to investigate the biological changes that occur over a year of spaceflight, and the Twins Study – which Scott Kelly was also involved in – compared the physiological and psychological differences that occur in space and on Earth (Garrett-Bakelman et al., 2019). Through over half a century of research, NASA has identified and categorized spaceflight hazards into 5 distinct categories, summarized with the acronym “RIDGE”: Space **R**adiation, **I**solation and Confinement, **D**istance from Earth, **G**ravity fields, and Hostile/Closed **E**nvironments (NASA, 2019a) (Table 1). In this section, we provide a brief overview of the ailments associated with each hazard, as well as existing strategies for the management of these clinical conditions. Distance from Earth is omitted in this discussion as it does not directly cause a clinical condition. An extensive discussion on the underlying pathophysiology and clinical studies on these spaceflight-associated medical ailments is outside the scope of this review. Therefore, readers interested in these are encouraged to refer to other review papers enumerated in the following sub-sections.

**Table 1:** Summary of spaceflight hazards and their associated clinical conditions and management strategies.

Spaceflight hazard	Clinical conditions	Management strategies	Possible medications	References
Space radiation	Degenerative diseases (e.g. cardiovascular diseases and cataracts)	Radiation shielding, radiation & health monitoring	Antioxidants⁎	(Fang et al., 2002; Gómez et al., 2021)
	Cancer	Radiation shielding, radiation & health monitoring	Antioxidants⁎	(Gómez et al., 2021; Montesinos et al., 2021)
	Changes in the central nervous system	Radiation shielding, radiation & health monitoring	Antioxidants⁎	(Cucinotta et al., 2014; Gómez et al., 2021)
Isolation and confinement	Behavioural changes	Gardening and journaling	Antipsychotics (aripiprazole and ziprasidone), anxiolytics (diazepam and lorazepam)	(Friedman and Bui, 2017; Odeh and Guy, 2017)
	Sleep problems	Light technologies, actigraphy	Hypnotic drugs (zolpidem, zaleplon), melatonin	(Friedman and Bui, 2017; Zivi et al., 2020)
	Fatigue	10-min self-test of vigilance and attention		(Basner et al., 2011)
	Decline in mood	Journaling, virtual reality sessions	Antidepressants (sertraline and venlafaxine)	(Friedman and Bui, 2017; Lyons et al., 2020)
Gravity fields	Muscle atrophy	Aerobic and resistive exercise, software-generated workout partners		(Feltz et al., 2016; Gao and Chilibeck, 2020)
	Bone loss	Aerobic and resistive exercise, software-generated workout partners	Bisphosphonate alendronate, supplements of calcium, vitamin D and K, omega-3 fatty acids, and proteins	(Feltz et al., 2016; Gao and Chilibeck, 2020)
	Spaceflight-associated neuro-ocular syndrome (SANS)	Compression cuffs, eye examinations	Supplementation with B vitamins⁎	(Petersen et al., 2019; Smith and Zwart, 2018)
	Space adaptation syndrome (SAS)	Minimizing vigorous head movement and activities	Antihistamines and anticholinergics (scopolamine and promethazine), stimulants (modafinil, caffeine)	(Estrada et al., 2012; Heer and Paloski, 2006)
Hostile/Closed Environments	Altered immune systems		Probiotics and prebiotics	(Turroni et al., 2020)
	Celestial dust exposure	Air filters		(Kobrick and Agui, 2019)
	Exposure to contaminants	Air filters, regular swabs to monitor microbial population, water treatment, flu vaccination		(Checinska Sielaff et al., 2019)

⁎ Suggested medications.

### 2.1 Space radiation

**Fig. 1:**
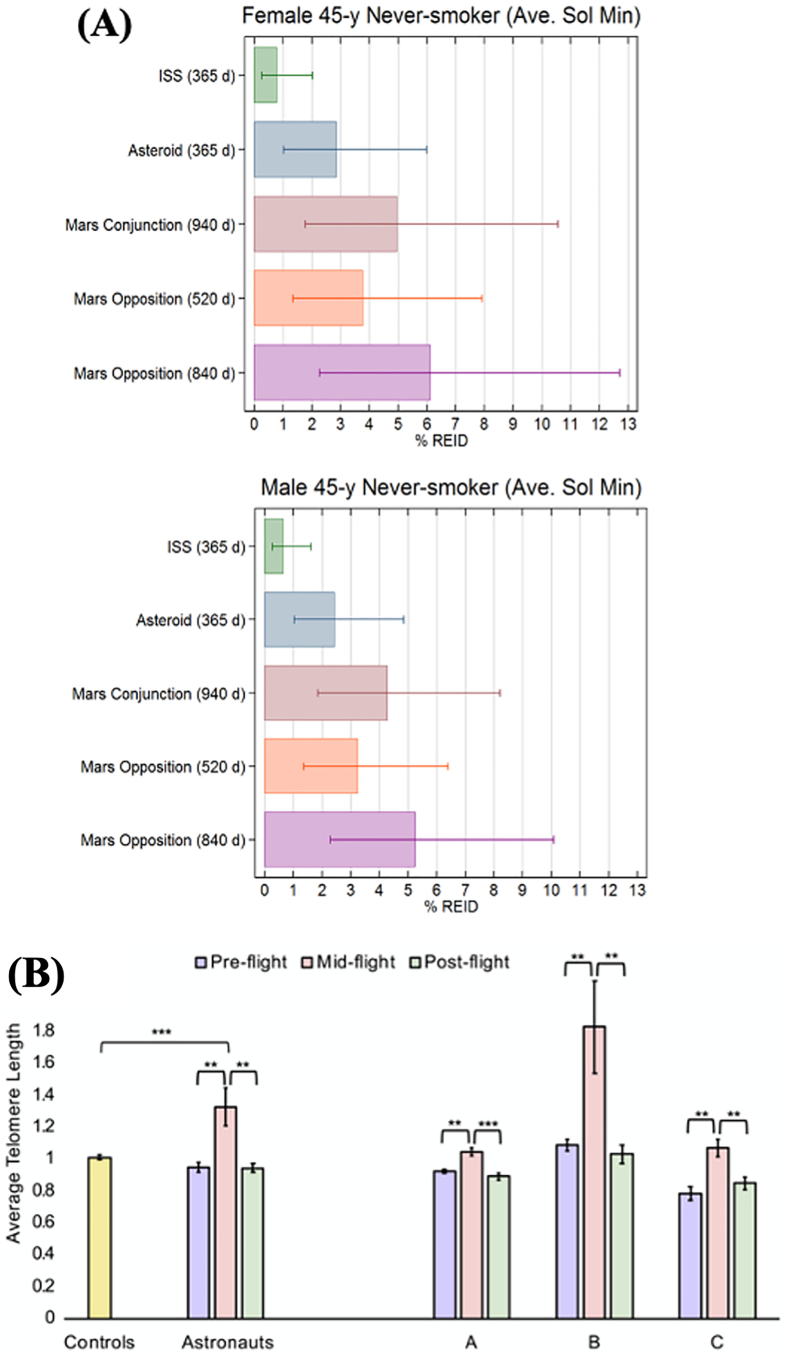
(A) Comparison of risk of radiation induced death (% REID) from circulatory diseases and cancer for different space missions in 45-year-old female and male never-smokers. Reproduced with permission from (Cucinotta et al., 2013) (B) Comparison of average telomere length of three crewmembers (*A, B,* and *C*) before, during and after spaceflight. Reproduced with permission from (Luxton et al., 2020).

Beyond Earth’s protective magnetospheres, humans are exposed to increased levels of radiation, primarily contributed by low dose-rate galactic cosmic rays and intermittent solar particle events (Afshinnekoo et al., 2020; Cucinotta and Durante, 2006; Cucinotta et al., 2013). Exposure to ionizing radiation inflict DNA double-strand breaks and oxidative damages, increasing the risk of carcinogenesis, degenerative diseases, and central nervous system effects (Cucinotta et al., 2014; Luxton et al., 2020) (Fig. 1). Degenerative diseases associated with elevated radiation exposure include cardiovascular diseases and cataracts. Epidemiological evidence has shown a significant increase in radiation-induced cardiovascular disease (RICVD), including myocardial infarction and stroke, at radiation doses as low as 0.5 Sv (Hughson et al., 2018). Other in-flight cardiovascular risks, such as arrythmias, also remain a concern. Evidence have also suggested an increased risk of cataracts because of increased radiation exposure in space (Chylack et al., 2009; Cucinotta et al., 2001). While the underlying aetiology remains unknown, radiation-induced cataracts is believed to be due to genetic damages in lens epithelial cells or from alterations of cortical lens fiber cells.

Acute and chronic effects of space radiation on the central nervous system (CNS) is also a concern. These are exacerbated by detrimental psychological and behavioural health inflicted by the long-term isolation and confinement that astronauts experience. Acute CNS effects include impaired motor function, altered neurocognitive function, and neurobehavioral changes, while chronic effects may include more sinister pathologies such as Alzheimer’s disease and dementia.

Oxidative stress induced by ionizing radiation, and aggravated by hypoxia and microgravity, is also linked to mitochondrial dysregulation. This could lead to cell death and mutations, disturbance of the expression of critical proteins, altered cellular functions and carcinogenesis (Azzam et al., 2012). Nutritional supplementation with exogenous antioxidants, such as vitamin E and C, polyphenols and flavonoids, could offer additional protective effects against free radicals (Guan et al., 2004; NASA, 2012). Moreover, the study of extremotolerant organisms, such as the tardigrade, has revealed an unique damage suppressor protein that protects the organism against damage from free radicals produced by ionizing radiation (Westover et al., 2020). The protective effect of this protein could potentially be used to mitigate oxidative stress damage resulting from chronic exposure to space radiation. Space radiation is also linked to numerous epigenetic changes, including altered methylation patterns and telomere lengths (Garrett-Bakelman et al., 2019; Luxton et al., 2020) (Fig. 1B). These findings highlight the importance of monitoring each crew member to assess their aging and disease risk, as well as their general health, as a means of developing personalized countermeasures based on each astronaut’s needs on future space missions.

In summary, the deleterious biological effects of pervasive radiation in space are significant, yet existing knowledge on their underlying mechanism is markedly lacking. More inquisitive readers may refer to the following reviews for further discussion on the existing evidence and mechanistic postulations of space radiation-induced pathologies (Hughson et al., 2018; Patel et al., 2020). Presently, the main proposed countermeasure to protect astronauts from radiation is to limit their exposure by including shields made of materials with high hydrogen content (e.g. polyethylene) that block ionizing radiation on spacecrafts. Other approaches involve the use of natural or synthetic lightweight compounds that absorb radiation and protect living systems from it, such as melanin (Dadachova and Casadevall, 2008). The combination of melanin with selenium, another compound that has shown protective properties against radiation damage (Shimazu and Tappel, 1964), led to the synthesis of selenomelanin (Cao et al., 2020), a compound that absorbs X-rays more efficiently compared to melanin. The discovery of more compounds like selenomelanin could help protect humans on long-term missions to Mars or beyond.

### 2.2 Isolation and confinement

**Fig. 2:**
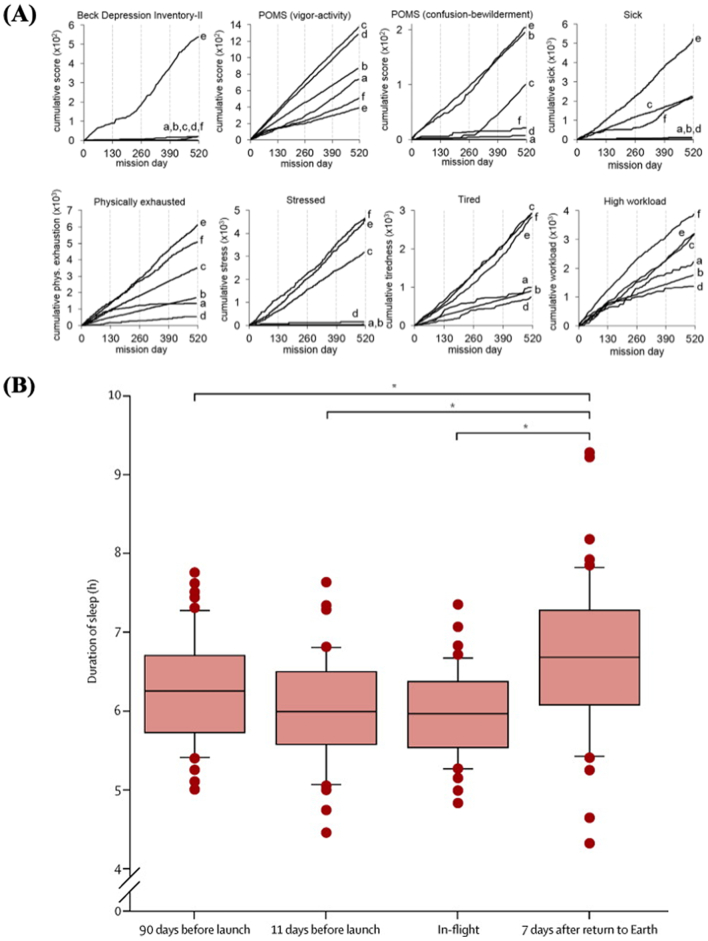
(A) Self report scores on reactions to confinement by crewmembers (identified by lower case letters a-f) on the Mars500 simulated mission. Measures of psychological distress include the Profile on Mood State (POMS), which is a short form comprising a list of 37 adjectives for which crewmembers indicated the degree each described themselves. Reproduced with permission from (Basner et al., 2014). (B) Average duration of sleep before, during, and after shuttle mission. Reproduced with permission from (Barger et al., 2014)

Human psychology is significantly altered in situations of confinement and isolation. In light of long-duration space exploration missions, behavioral health risks need to be addressed, as they could have a large impact on the success of a mission (Pagel and Choukèr, 2016). Earth-based studies that simulate the isolation and confinement of space in analogue environments, such as Mars500 (Basner et al., 2014) and Antarctic expeditions (NASA, 2016; Suedfeld, 2018), show that social isolation might lead to neurological deficits (Leser and Wagner, 2015), immune dysregulation (Crucian et al., 2014) and increased anxiety (Wallace et al., 2009) (Fig. 2A). Another major problem is distance from Earth, which causes psychological stress on crewmembers and could disrupt team dynamics (Bell et al., 2019; Landon et al., 2017). Existing management strategies include antidepressants (sertraline and venlafaxine), antipsychotics (aripiprazole and ziprasidone) and anxiolytics (diazepam and lorazepam) that are included in the ISS medical kit (Friedman and Bui, 2017).

The stress of prolonged isolation and confinement, coupled with persistent noise and different dark/light cycles, also causes disruption to astronaut’s circadian rhythm. Sleep deprivation and fatigue remain common problems among astronauts aboard the ISS (Barger et al., 2014; Wu et al., 2018) (Fig. 2B). Alterations in circadian rhythms are related to numerous health conditions such as inflammation and immune system dysregulations (Gachon et al., 2018), and is also critical for gastrointestinal function, as the microbiome itself exhibits circadian rhythms (Kuang et al., 2019). In view of long-term missions to Mars or other planets, the need for sleep aids is clear (Wotring, 2012). Zolpidem and zaleplon are the frequently used medications on spaceflights (Barger et al., 2014). However, the use of hypnotics with long half-lives or the repeated dosing at night could be dangerous in the event of an emergency that requires the complete focus of the crew (Wotring, 2012). Thus, it could be interesting to use drugs such as melatonin to help re-establish the circadian rhythm and reduce dependence on hypnotics (Li et al., 2019).

### 2.3 Gravity fields

Microgravity is the condition of near weightlessness associated with spaceflight. This unique environment induces pronounced changes to the human body, some of which are maladaptive. Foremost, 60–80% of astronauts experience space adaptation syndrome (SAS), also known as space motion sickness (SMS), within the first couple of days in orbit (Hodkinson et al., 2017). This occurs due to conflicting sensory information from the visual system and the information from the vestibular and proprioceptive systems (Heer and Paloski, 2006; Wotring, 2012). Current management strategies involve minimizing vigorous head movement and activities. Prophylactic treatment with antihistamines such as promethazine, occasionally accompanied by stimulants like modafinil to reduce lethargy, is also effective (Estrada et al., 2012; Wotring, 2015).

The elimination of gravitational loading also leads to compensatory changes in the cardiovascular system, with fluids shifting towards the head and thorax, otherwise known as cephalad fluid shift (Hughson et al., 2018). This leads to noticeable physiological changes, manifested as ‘puffy face’ associated with facial oedema and ‘chicken legs’ due to reduced leg volume. Extended durations in Space can also result in spaceflight-associated neuro-ocular syndrome (SANS) due to the increased intracranial pressure. These lead to visual disturbances that affect the crew’s ability to perform their tasks (Lee et al., 2020a). Potential countermeasures proposed to SANS include the use of a negative pressure device that pulls fluid away from cranial compartments (Petersen et al., 2019) and supplementation with B vitamins (Smith and Zwart, 2018). As SANS shares some features with idiopathic intracranial hypertension, acetazolamide could be a treatment option (Wall et al., 2014). However, this drug increases the risk of kidney stones and therefore is not recommended in people at risk of kidney stones such as astronauts, due to the continuous bone resorption and formation that occurs during spaceflights (Smith et al., 2015; Wotring, 2012). Due to the prevalence of SANS amongst crewmembers, eye examinations are now common practice on the space station (NASA, 2020a).

**Fig. 3:**
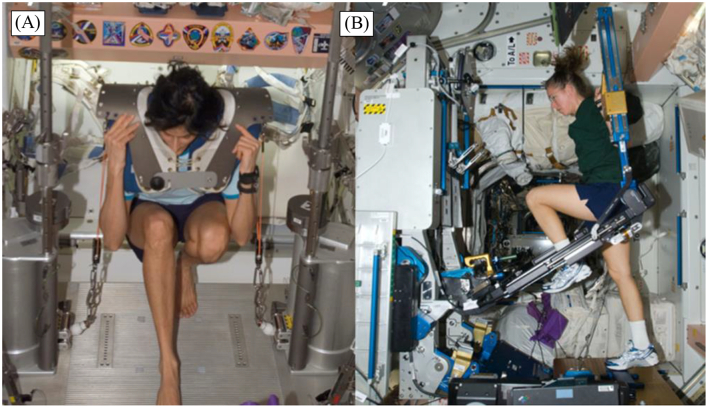
Images of astronaut exercising on (A) Interim Resistive Exercise Device (iRED) (donning squat harness pads), and (B) Advanced Resistive Exercise Device (ARED). Reproduced with permission from (Patel et al., 2020).

Gravitational unloading also deconditions the musculoskeletal system, resulting in bone loss and muscle atrophy during spaceflights, exacerbated by reduced food intake by the crew during missions (Smith and Zwart, 2008). Bone resorption increases in microgravity regardless of physical activity, while bone formation remains unchanged or decrease without resistance exercise (Grimm et al., 2016; Smith et al., 2015; Smith et al., 2012). This negative balance between bone resorption and formation results in bone loss and an increased renal stone risk during spaceflight (Smith et al., 2015). The combination of bisphosphonate alendronate with resistive exercise countermeasures using the Advanced Resistive Exercise Device (ARED) or the Interim Resistive Exercise Device (iRED) (Fig. 3), have proven to be effective in preventing or attenuating crewmembers’ mean decreases in bone mineral density (Sibonga et al., 2019). Additionally, space agencies have also provided the crew with supplements of calcium, vitamin D and K, omega-3 fatty acids and proteins, in an effort to mitigate bone loss (Gao and Chilibeck, 2020; Smith et al., 2012). On the other hand, skeletal muscle atrophy, especially in the lower body, occurs as the absence of gravitational loading forces effectively renders lower leg muscles redundant (Afshinnekoo et al., 2020; Fitts et al., 2001). Some studies have identified altered levels of proteins critical to muscle regeneration in astronauts, such as myostatin, activin A, and certain cytokines (e.g. IL-6, IL-10, IL-1ra), which may represent potential targets for pharmacological interventions (Gertz et al., 2020; Lee et al., 2020b; Smith et al., 2020). At present, resistance exercise is the main countermeasure for preventing muscle atrophy and bone loss.

Astronauts also commonly experience lower back pain due to microgravity (space adaptation back pain), arising because of a loss of spinal curvature and spinal elongation (Green and Scott, 2018). Headaches are also common, associated with microgravity-induced cephalad fluid shifts and exposure to high CO_2_ levels on the ISS (Law et al., 2014; Lawley et al., 2017). Ibuprofen is the general analgesic of choice, although the use of other pain relievers such as aspirin, acetaminophen and hydrocodone has also been reported (Stingl et al., 2015).

### 2.4 Hostile/closed environments

The ecosystem within the spacecraft is well-regulated to shield crewmembers from the potentially hazardous substances in space, such as celestial space dusts and pathogenic microorganisms. Examples of these control measures include Analyzing Interferometer for Ambient Air (ANITA) and active thermal control systems (Archive, 2008). Astronauts’ stress hormone levels are also elevated, and their immune system is known to be altered by spaceflight. These stressors could not only increase the crewmembers’ susceptibility to illnesses but might also lead to the development of autoimmune diseases.

There is increasing evidence that maintaining a healthy and diverse population of microbes is essential for a healthy body. However, microbial intake on space missions is severely deficient, due to the absence of microbes in astronauts’ food to extend shelf lives and the heavily filtered air that astronauts breathe to minimize the risk of infections. Additionally, astronauts are exposed to a number of stressors that can cause imbalances in their microbiomes during the course of a mission, potentially negatively impacting their health (LaPelusa et al., 2021). Astronauts that spent six to twelve months on the ISS have shown significant changes in intestinal, skin, nose and tongue microbiome during the space mission (NASA, 2011; Voorhies et al., 2019). However, the impact of microbiome shifts on crewmembers’ health needs further investigation to fully appreciate its consequences. The use of probiotics and prebiotics to counteract microbiome dysregulation during space missions has been proposed based on their recognized benefits for gut health, either added to food or as a supplement (Turroni et al., 2020). Other approaches include incorporating vegetables into the astronauts’ diet, as fiber-rich diets promote the growth of butyrate-producing bacteria, which have a positive impact on overall human health (Voorhies and Lorenzi, 2016). Although space farming is still in its infancy, it might be possible in the future to grow fruits and vegetables in other planets or during long-term missions (Berkovich et al., 2004; Moroz et al., 2020).

## 3 Altered clinical effects of medicines in space

**Fig. 4:**
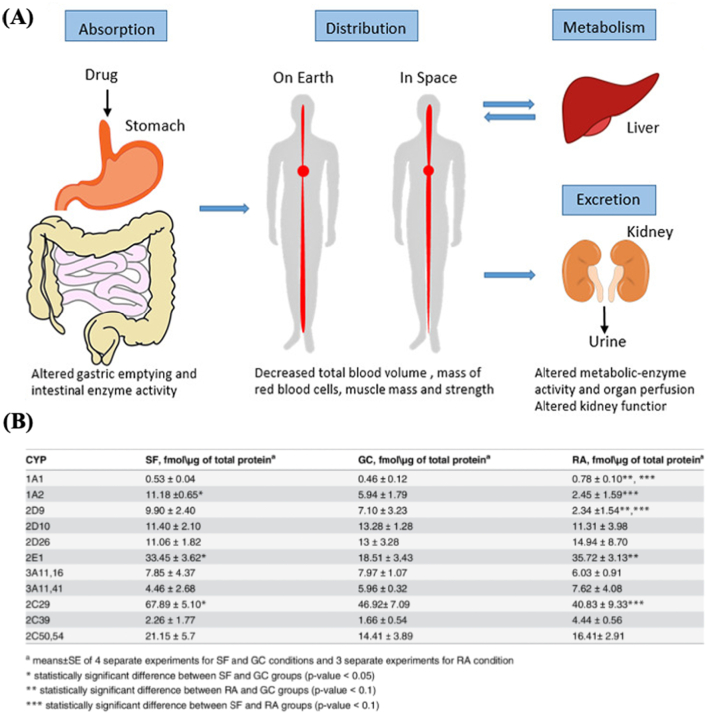
(A) Effects of the spaceflight environment on pharmacokinetics of drugs. Reproduced with permission from (Kast et al., 2017). (B) Table showing the differing concentrations of cytochrome P450 enzymes in mice exposed to 30 days of space flight (SF), the ground control (GC), and the recovery group (RA). Reproduced with permission from (Moskaleva et al., 2015).

Due to the significant physiological changes that occur under microgravity, drug absorption, distribution, metabolism, and excretion (ADME) can be modified during spaceflight (Fig. 4A). For instance, drug absorption is affected by an altered gut microbiome, or by delayed gastric emptying caused by space motion sickness. On the other hand, dehydration, muscle atrophy, and bone loss can modify the distribution and excretion of drugs, thereby potentially modulating drug efficacy and adverse effects (Kast et al., 2017). Moreover, it has been observed that the activities of various enzymes were altered in animals exposed to microgravity. Notably, several intestinal digestive enzymes, including adenosine triphosphatase and glucose-6-phosphatase, have elevated activities during space flights (Graebe et al., 2004). Mice exposed to 30 days of space flight and microgravity were also found to have significantly increased levels of CYP2E1, CYP1A2, and CYP2C (Moskaleva et al., 2015) (Fig. 4B). Elevation of these drug metabolizing CYP enzymes could lead to accelerated metabolism of drugs and consequently an attenuation of pharmacological effects. Adverse effects associated with these changes must be explored and defined, as they will have a major impact on the efficacy and safety of the medications administered to astronauts. The pharmacokinetic and pharmacodynamic changes in space have been reviewed elsewhere, which we direct curious readers towards for further details that are otherwise not discussed in this review (Eyal, 2020; Kast et al., 2017).

**Fig. 5:**
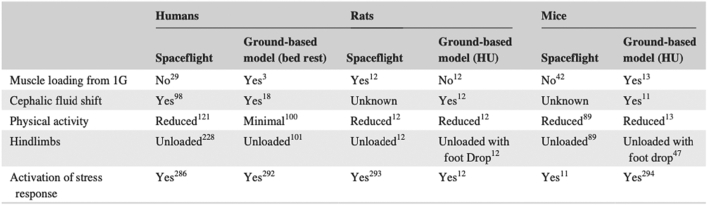
Table showing the difference in muscle unloading characteristics between human and rodent models. Reproduced with permission from (Qaisar et al., 2020).

Despite more than five decades of manned spaceflight, the data available on pharmacokinetics in space is limited and is based on sporadic evidence. To date, few clinical studies have evaluated drug pharmacokinetics in astronauts during spaceflight, and these have been limited to saliva samples (Boyd et al., 2009; Cintron et al., 1987a; Cintron et al., 1987b; Kovachevich et al., 2009; Putcha and Harm, n.d.; Putcha and Kovachevich, n.d.). Several limitations complicate the interpretation of results from these studies. For example, drug concentration measurements were made in saliva (saliva-to-blood ratios in space are unknown, and this could introduce errors), and were sampled from a small number of subjects. There was also significant variability in sampling time, food consumption, and route of administration. Nevertheless, existing evidence point towards altered drug disposition during spaceflight compared to measurements on Earth (Eyal, 2020). Rodent models and bed rest models are often used to simulate physiological changes seen during microgravity, as they can mimic microgravity-induced fluid changes (Mulavara et al., 2018; Qaisar et al., 2020). Nonetheless, human studies are paramount for deepening our understanding, as animal models fail to account for other confounding variables of spaceflight (Blue et al., 2019a) (Fig. 5). In general, spaceflight-associated alterations in pharmacokinetics and pharmacodynamics remain a vastly unexplored field. The sparse reports on the subject make astronauts an ‘orphan’ population with respect to the safety and efficacy of drugs. Furthermore, existing data are solely derived from astronauts, who have passed stringent health and medical screenings. Consequently, these reports might not be representative of an average human being; therefore, clinical trials on civilians in space is necessary to support the advent of space tourism.

To ensure the safety and efficacy of drugs administered in space, it might be pertinent to conduct clinical trials in space. However, given the small number of people who travel to space, such randomized controlled trials will likely be statistically underpowered. Recommendations on the design of clinical trials for rare diseases published by the International Rare Diseases Research Consortium (IRDiRC) could be applied to increase the efficiency of clinical trials in space (Day et al., 2018). These include reducing bias and increasing efficiency by using analysis of covariance instead of “change from baseline” analyses, using longitudinal data (analysis of repeated measurements) as opposed to change score analysis to potentially reduce sample size, and using composite endpoints to increase statistical power. Establishing a robust pharmacovigilance system dedicated to monitoring adverse events and drug problems encountered in space would also benefit our understanding on the alterations to pharmacokinetics and pharmacodynamics (Blue et al., 2017). It would be interesting to be able to predict changes in the pharmacokinetics or in the safety of drugs using pharmacokinetic modelling tools currently available. However, existing pharmacokinetic modelling tools are not complex enough to predict pharmacokinetic changes based on, for example, how many days in microgravity and the individual has been. Instead, machine learning models could be developed to predict drug concentrations based on changes to baseline physiological parameters. For this to happen, sufficient data on crewmembers’ health and corresponding drug pharmacokinetic parameters must be available.

While feasible options exist for conducting clinical trials in space, there is ambiguity over who the stakeholders might be. To mitigate the small patient pool and bolster statistical power, it might be necessary to pool clinical data on every human being in space, regardless of nationality. Therefore, unlike conventional clinical trials, government agencies and national regulatory authorities might not be efficient in this case, especially given the lack of regulatory harmonization (Crow et al., 2018). Instead, an international regulatory agency for medicines in space (Space Medicines Agency) could be established in the future to facilitate these studies and enforce a system of pharmacovigilance. This agency could adopt a governance structure akin to the World Health Organization (WHO), whereby a seasoned regulatory affairs official is appointed by every Member State to serve on the Executive Board. This regulatory agency will also need to incentivize pharmaceutical companies to contribute to the clinical trials in space, as the small patient population (and therefore addressable market) dwindles the direct return on investments for these companies. This incentives program might adopt existing strategies such as monetary subsidies, market exclusivity extensions, and priority review vouchers (Selker and Pardes, 2018). Alternatively, considering the niche and challenge, it might be imperative to the implement an entirely novel incentive program to entice companies. Through such international collaboration involving both the public and private sector, our understanding of the altered clinical effects of medicines in space can be deepened, allowing us to better support our ambitions in deep space.

## 4 The current state of medicines in space

### 4.1 Current medicine storage and management strategies

At present, medical supplies on the ISS are regularly replenished by cargo resupply services missions, ensuring medicines are kept and used within their expiration dates. However, this would not be possible for deep space missions to Mars or other planets given the significantly longer distance and duration of travel (Wotring, 2012). Instead, there are two possible scenarios: the use of stored medicines that can maintain their shelf-lives throughout the entire mission, or the on-board preparation of medicines using stored excipients and drugs.

Medical kits have been carried on board space shuttles since the beginning of manned space flights. Currently, the ISS medical kit includes around 190 medications, in addition to bandaging supplies, catheterization items, diagnostic devices and microbiological test kits. Commonly used medicines are separated from specialized medicine packs for emergencies and from drugs used in research studies (Wotring, 2012). However, medications are susceptible to degradation over time, especially if they are exposed to light, humidity, or oxygen. Although the environmental conditions on the ISS appear to be adequate for medication storage, other unique environmental factors of spaceflight (e.g. radiation exposure, microgravity) could affect chemical stability and degradation of drugs (Du et al., 2011). Moreover, solid dosage forms are generally repackaged into polyethylene bags to reduce bulk of medical kits, reducing their shelf lives as they are deprived of their protective packaging (Putcha et al., 2016). Until now, frequent resupply missions to the ISS have overlooked studies on degradation, storage, and the impact of radiation and microgravity on drug stability. Additionally, collecting data on medication use, efficacy and side effects, as well as pharmacokinetics and pharmacodynamics has not been a priority of NASA’s Human Research Program (Blue et al., 2019b).

Among the environmental factors of space travel, chronic exposure to radiation is possibly the main additional contributor to instability of pharmaceutical products. Although gamma radiation and X-rays are commonly used to sterilize pharmaceuticals on Earth (Armenante and Akiti, 2019), space radiation differs from the radiation used in these processes in the type of radiation, dose, dose-rate and exposure time. Direct ionization is radiation that strikes a target molecule and can cause the rupture of chemical bonds and the destruction of polymer structure (Kempner, 2001). On the other hand, when radiation hits water instead of a target, the radiation generates radiolitic products that can diffuse and damage a target molecule within range (Nisar et al., 2016). This type of radiation is called indirect ionization. Dry materials do not have solvent radiation products, and in frozen materials, although radiation products are generated as in the liquid state, the diffusion constant is much lower (Kempner, 2011). Thus, liquid pharmaceuticals are often considered less stable than solid or powdered dosage forms, given the formation of radical oxygen species from the breakdown of water when it is irradiated (Blue et al., 2019b). Although pharmaceuticals in the solid state undergo almost no indirect ionization, they are not exempt from damage caused by direct radiation on individual molecules (Ambroż et al., 2000; Sarcan and Ozer, 2020).

Currently, the extent to which space radiation affects drug stability, produce toxic by-products, or alter drug ingredients is not clearly established. Preliminary studies and simulation models to evaluate these possibilities are limited and therefore it is not possible to reach a conclusion with the available data (Kim and Plante, 2015). Some of these studies were conducted on a variety of pharmaceuticals stored on the ISS and compared to ground controls. In one of the studies, 35 formulations stored on the ISS were analyzed after 28 months in space, and the results suggested that there may be differences in the degradation rate and potency of formulations on the ISS compared to those stored on Earth (Du et al., 2011). Moreover, some formulations appeared to be more sensitive than others, indicating that certain chemical characteristics may influence stability of drugs exposed to the space environment. In a later study, expired drugs that had been stored on the ISS for 550 days were returned to Earth for analysis (Wotring, 2016). Nine medications were chemically analyzed and although no unusual degradation products were identified, three of the drugs failed to meet purity requirements. Unfortunately, this study lacked ground controls, so no conclusions can be drawn regarding the possible effects of space travel on medication stability. Alterations were also found in multivitamins flown to space and stored on Earth when compared to time-zero controls (Chuong et al., 2011; Zwart et al., 2009). The alterations found appeared to be more chemical than physical in nature, however no convincing evidence of degradation was found in any study.

The type of packaging materials could help prevent radiation-induced degradation of drugs. Low atomic number materials, such as hydrogenous materials (e.g. polyethylene) are known to be more effective than high atomic number materials, such as aluminum, in reducing exposure to space radiation (Badhwar and Cucinotta, 2000; Shavers et al., 2004). This is because the production of neutrons and other secondary particles reduces as the atomic number of the material decreases. As such, hydrogen is the best material. The development of novel hydrogen-rich materials with better radiation shielding capabilities could help protect drugs from space radiation on future space missions (Iguchi et al., 2018; Naito et al., 2020). In one study, 80% of natural biomaterials, such as collagen and silk, were found to be chemically crosslinked by space radiation after being flown for 18 months on the ISS (Hu et al., 2013). The use of protein-inorganic composites or pre-crosslinked protein materials could help reduce the impact of radiation on them and could also be used as protective shields to coat medicines.

Cryogenic temperatures (around 100 K) are known to protect biological samples from ionizing radiations employed in X-ray crystallography and electron microscopy (Meents et al., 2010). At these temperatures, the samples tolerate a significantly higher radiation dose, however, the optimal cryogenization temperature to minimize damage is still unknown (Chinte et al., 2007; Meents et al., 2007). Within the pharmaceutical field, some studies showed that cooling suspensions of morphine and insulin before sterilization with gamma rays or X-rays improved the stability of the drugs during the process (Maquille et al., 2008; Moyne et al., 2002; Soboleva et al., 1981). Therefore, one strategy to protect medicines from space radiation could be storing them at cryogenic temperatures, which is easily attainable due to the lower temperatures in space (around 3 K). However, this might not be suitable for all pharmaceuticals, and further research is needed on this topic by exposing drugs to ionizing radiation at different temperatures. The post-exposure analysis could shed light on differences in drug stability differences as a function of temperature (Mehta and Bhayani, 2017).

Microgravity is another source of potential instabilities for pharmaceuticals in space. In some liquid formulations, such as suspensions and emulsions, the absence of gravitational forces is beneficial as it reduces the chances of particle sedimentation or coalescence. However, the lack of gravity also prevents the elimination of air bubbles in injectable medications. The air bubbles could be eliminated from the formulations before being sent to space, however, as drugs are often more stable as solids than liquids, it is preferred for parenteral medications to be stored in their freeze-dried form to extend their shelf life. Therefore, it is necessary to understand how these air bubbles can be eliminated in space (Mehta and Bhayani, 2017).

### 4.2 Challenges of adopting conventional medicinal manufacturing in space

**Fig. 6:**
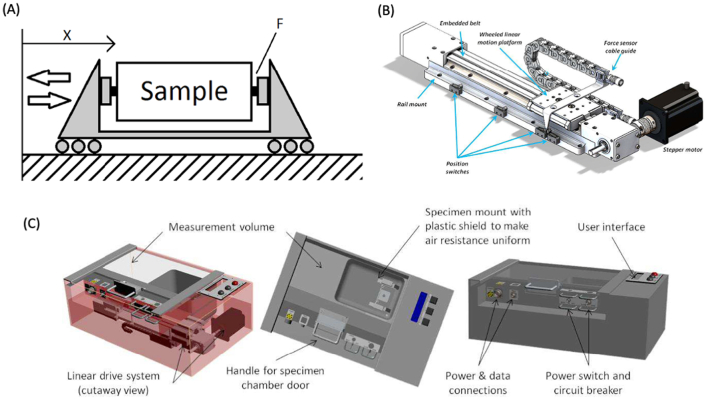
(A) Figure showing the working principle of the ZGMMD. The sample moves at a pre-defined acceleration (x) and force sensors measures the force applied by the sample (f), (B) Schematic of ZGMMD Phase II, (C) 3D views of ZGMMD Phase II design. Reproduced with permission from (Morrow et al., 2015).

To create just-in-time medicines in space, there is a need to explore alternative manufacturing methods. This is because conventional drug manufacturing involves the use of heavy machinery, such as the tablet press and capsule filling machines, which are hardly feasible given the constrained space and payload limits of a spacecraft (Domokos et al., 2020). There is also the fundamental issue of how drugs and excipients can be weighed in microgravity. Medicines are made up of a mixture of active pharmaceutical ingredient(s) and excipients, such as glidants, lubricants, binders, and disintegrants. On Earth, components are individually weighed or measured, usually in the form of powders or liquids respectively, and subsequently mixed to form a homogenous powder/liquid mixture. Both parameters – volume and weight – are difficult to measure under microgravity. While weight cannot be measured in the absence of gravity, astronauts can measure mass using the Zero Gravity Mass Measurement Device (ZGMMD) (Fig. 6). The device works according to Newton’s second law, applying a known acceleration to a sample and measuring the resultant force (Shimada and Fujii, 2012). Thus, it would be possible to measure the exact dose of a drug, although the precision of the device should ideally be in the milli- or even micro-gram range.

Another option could be the preparation of liquid formulations akin to how freeze-dried foods are reconstituted in space (NASA, 2021). First, the exact amount of powdered drug and excipients are weighed on Earth and packaged in a drink container sealed on top with a one-way plastic valve. Once in orbit, the one-way valve allows water to be directly injected, reconstituting the formulation immediately prior to administration. Depending on the choice of excipients, the resulting preparation could have a gel-like consistency similar to sports gels used by athletes, which are easy to consume and have a pleasant taste. As formulations are reconstituted only immediately before administration, the issues of instability in standard liquid dosage forms, such as precipitation, flocculation, and hydrolysis, are obviated. However, as these must be pre-made on Earth, it is conceivable that medicines made in this way would adopt a one-size-fits-all approach towards dosing, rather than tailoring the dose according to the crew member’s unique microgravity-induced physiological alterations. Their stability after reconstitution must also be assessed in case the stability of the drug and the excipients have been compromised during space travel. Moreover, the packaging increases the space needed to transport the medications and the total weight of the medical kit.

The problems discussed above and the existing gaps in knowledge on the behaviour of drugs stored in space, make it pertinent to establish systems to support the on-demand production of drugs in space, assuring their stability while saving storage space and reducing the payload.

## 5 Innovative manufacturing methods for medicines

**Fig. 7:**
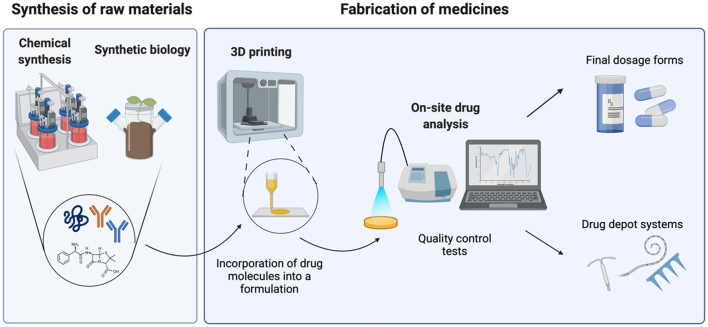
Overall schematic of alternative synthesis and manufacturing strategies for the on-site production of medicines in space. Created with BioRender.com.

Alternative processes and their corresponding machinery must be adapted to work in microgravity and in varying degrees of gravitational force. Strategies should not be limited to the preparation of dosage forms but encompass the entire drug manufacturing process, starting from the chemical synthesis of active pharmaceutical ingredients. In this section, we discuss emerging technologies that may be suited for extra-terrestrial application, covering both the synthesis of raw materials and the manufacturing of the final medicinal product (Fig. 7).

### 5.1 On-demand synthesis of raw materials

**Fig. 8:**
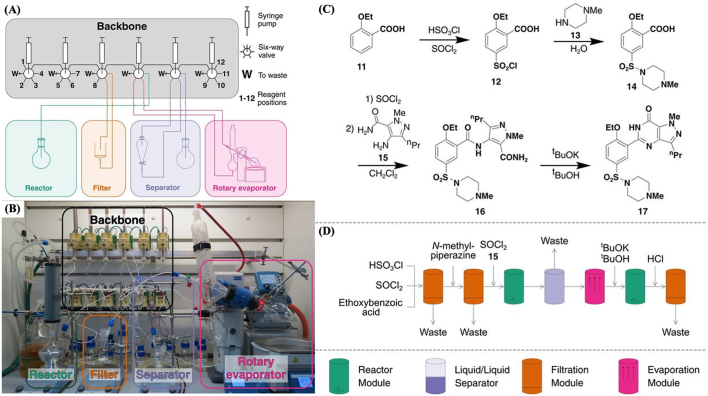
(A) Schematic representation of the Chemputer highlighting the four modules used (reactor, filter, separator, and rotary evaporator). (B) Photograph of one Chemputer setup with the modules highlighted in correspondence to the schematic. (C) Chemical reaction and (D) schematic of sildenafil synthesis. Reproduced with permission from (Steiner et al., 2019).

Numerous approaches have been investigated and developed for on-site synthesis of therapeutic molecules, such as chemputing, the Defense Advanced Research Projects Agency’s (DARPA) Pharmacy on Demand (PoD) and Biologically derived Medicines on Demand (Bio-MOD) system, and synthetic biology platforms. Chemputing represents a novel method of synthetizing drug molecules using a modular chemical-robot system known as a Chemputer that could represent a breakthrough in pharmaceutical manufacturing (Fig. 8) (Steiner et al., 2019). Using a relatively small range of modular equipment comprising a reaction flask, a jacketed filtration setup, an automated liquid-liquid separation module and a solvent evaporation module, the Chemputing pioneers at the University of Glasgow were able to synthesize a variety of pharmaceutical compounds, such as rufinamide, diphenhydramine hydrochloride and sildenafil. They also developed a program, dubbed the Chempiler, to automate synthesis performed by the Chemputer. Through this approach, the researchers achieved isolated yields comparable to those achieved manually, specifically 58% for pure diphenhydramine hydrochloride (slightly lower than yield (68%) achieved manually), 46% for rufinamide (slightly higher than yield (38%) achieved manually), and 44% for sildenafil. This laboratory-scale synthesis robot would allow on-demand synthesis of molecules in an affordable and safe manner, with the use of a modular Chemputer and a software app (Cronin, 2019).

Perhaps the most prominent benefits of this technology would be found in hard-to-reach areas, such as deep space or disaster zones, where access to medicines is not assured. Healthcare professionals could create life-saving drugs simply by downloading the blueprint of the organic chemical, and the Chemputer would create the molecule in the same way as if it were following a cooking recipe. This also applies to newly discovered drugs; for example, if a new drug is developed on Earth, crewmembers on a space mission could download the computer code for the new molecule and create it on-board (University of Glasgow, 2018; Wood, 2020). By enabling the synthesis of a multitude of drugs from a few starting reagents, the portion of the payload assigned to medicines may be significantly reduced. Admittedly, there remains outstanding issues to be resolved, such as making all drug syntheses available online, reducing the size and complexity of the Chemputer, and subsequently adapting it for functionality in microgravity. However, the immense benefits that this technology could bring to healthcare by democratizing and decentralizing pharmaceutical manufacturing makes it worth investing in its development.

**Fig. 9:**
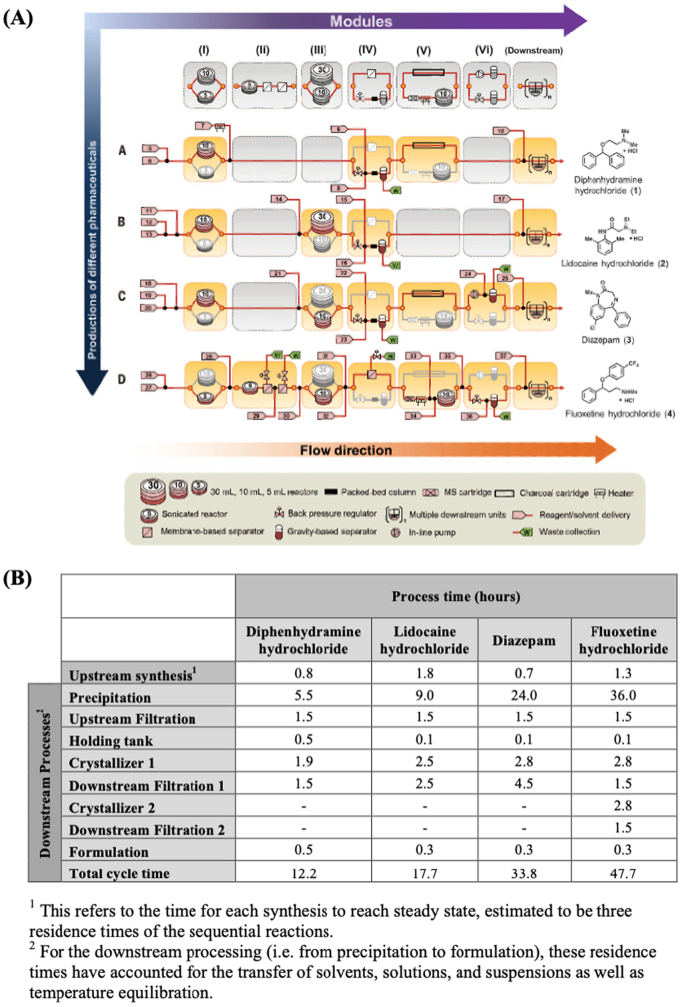
(A) Schematic representation of the reconfigurable system for continuous drug synthesis. At the top, the row represents the different modules; coloured modules are active, while grey boxes represent inactive modules. (B) Table showing the breakdown of process time for each API tested, Reproduced with permission from (Adamo et al., 2016).

The DARPA Battlefield Medicine program aims to develop miniaturized manufacturing platforms to synthetize pharmaceuticals on the battlefield to address specific medical needs as they emerge on the frontline (McClure-Begley, n.d.). The program includes two initiatives; Pharmacy on Demand (PoD) and Biologically derived Medicines on Demand (Bio-MOD), both with the aim of manufacturing therapeutic products on-demand (Arnold, 2019). The PoD initiative aims to create a reconfigurable and mobile device that combines both synthesis and final drug product formulation in the same unit (Group, n.d.). With this refrigerator-sized system (1.0 m x 0.7 m x 1.8 m (W x L x H)), hundreds of doses of four different active pharmaceutical compounds were synthetized from raw materials, namely diphenhydramine hydrochloride (82% yield and 4,500 doses per day), lidocaine hydrochloride (78% overall yield, 97.7% purity, and 810 doses per day), diazepam (94% yield and 3000 doses per day), and fluoxetine hydrochloride (43% yield and 1100 doses per day) (Fig. 9) (Adamo et al., 2016). The system could reproduce results within a standard deviation of 0.6–4.7% yield and was found to have no cross-contamination between runs. However, the system still needs to meet Good Manufacturing Practice (GMP) requirements to obtain approval from regulatory agencies.

**Fig. 10:**
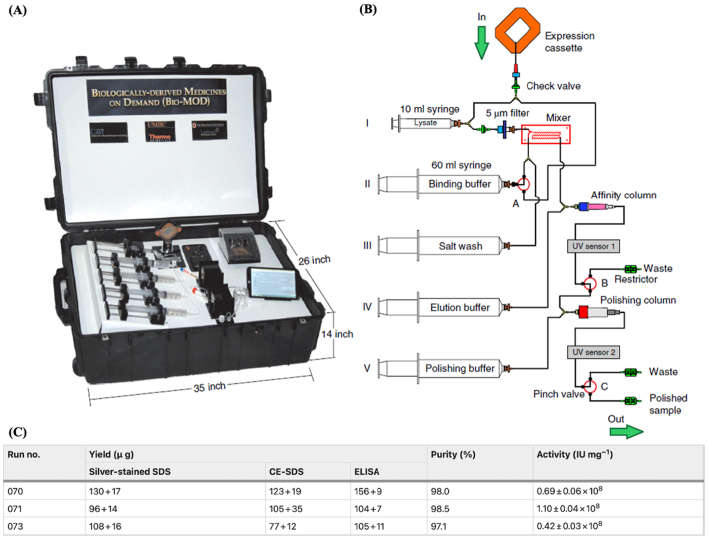
Biologically derived medicines on demand, Bio-MOD, a miniaturized platform for biologics manufacturing. (A) Photograph of the suitcase-sized platform showing the components. (B) Process workflow of therapeutic protein manufacturing. (C) Table showing yield, purity, and activity of G-CSF-His produced in the Bio-MOD over 3 runs. Reproduced with permission from (Adiga et al., 2018).

On the other hand, the Bio-MOD system is designed to produce therapeutic proteins in small doses at the point-of-care in less than 24 h (Fig. 10) (Adiga et al., 2018). The device includes a module for *in vitro* translation of target proteins from ribosomal DNA of reconstituted lyophilized Chinese hamster ovary cells and is coupled to another module for protein purification with integrated quality control (UMBC, n.d.). At present, the Bio-MOD system has been successfully used to synthetize His-tagged granulocyte-colony stimulating factor (G-CSF-His), glucose-binding protein, diphtheria toxoid DT5 and erythropoietin with satisfactory yield, purity, and activity. For instance, the activity of G-CSF-His produced by the Bio-MOD system was comparable to that of commercial equivalents (e.g. Amgen’s Neupogen and Sandoz’s Zarxio) which have specific activities of 1.0 ±  0.6 × 10^8^ IU mg^−1^ (Fig. 8C) (Adiga et al., 2018). However, as with the PoD system, compliance with GMP requirements is still necessary for the device to obtain regulatory approval before it can be clinically deployed.

Similarly, synthetic biology is an emerging discipline that aims to re-engineer organisms to produce high-value products such as protein therapeutics, biofuels, and bio-inspired materials. The ability to transform microorganisms to serve as biofactories brings new options for manufacturing biological drugs (El Karoui et al., 2019; Trosset and Carbonell, 2015). The creation of synthetic biology platforms capable of operating outside of well-controlled laboratory conditions could enable its deployment in areas with limited resources and variable storage conditions, such as remote military and space missions, for the on-demand production of protein-based drugs (Bendicho-Lavilla et al., 2022; Brooks and Alper, 2021). Given how promising this discipline is, NASA’s Translational Research Institute for Space Health (TRISH) has funded a project at the Massachusetts Institute of Technology (MIT) to develop a gastric resident device for *in-situ* production of drugs, called the “mother machine”. This drug delivery device manufactures and gradually releases drugs within the stomach after ingestion. Once the drug is released, the device disintegrates and passes through the gastrointestinal tract safely (Medical, n.d.). The “mother machine” uses bacterium (e.g. E. Coli) to produce and release three drugs – melatonin, caffeine and acetaminophen – commonly used to treat minor conditions such as sleep disorders and headaches.

In another TRISH-funded project, researchers at the University of California Davis are developing plant-based methods to produce protein-based pharmaceuticals in under 24 h for use in deep space missions (Pflueger-Peters, 2020). The researchers chose lettuce as their host plant as it had already been successfully grown on the ISS. Lettuces were infected with *Agrobacterium tumefaciens* containing a DNA sequence encoding the desired protein, which is subsequently transcribed and translated into the functional protein. They focused on producing three different therapeutic proteins — parathyroid hormone (PTH) for bone loss in microgravity, granulocyte colony-stimulating factor (GCSF) for radiation sickness, and an anti-fungal peptide (AAAS, Reports and Proceedings, 2022). This technique could enable on-demand drug manufacturing by allowing astronauts to grow plants that have been genetically modified to produce pharmaceuticals. Payload demand can be kept minimal as only the seeds of the modified plants need to be loaded. However, isolating the product from the plants remains necessary and would require additional machinery. Moreover, the platform must be operational in microgravity and with limited water resources (Brooks and Alper, 2021). Nevertheless, once isolated, the exact dose of the drug could be mixed with suitable excipients and the desired dosage form could be fabricated by means described hereon.

Emerging therapeutics such as siRNA-, mRNA-, and CRISPR-based therapeutics are poised to play a critical role in pharmaceutical care due to their ability to treat diseases at their genetic root. mRNA-based therapeutics are synthesized in cell-free systems, making their synthesis and purification process simpler than that of existing biotherapeutics (Sahin et al., 2014). CRISPR-based therapeutics, while presently still in clinical trials, could obviate the need for repeated administration, thereby significantly reducing the necessary medical payload. Therefore, nucleic acid-based and gene-editing based therapeutics could revolutionize pharmaceutical treatment in space. However, these expensive modalities are reserved for the most malignant of diseases. Small molecule drugs will likely continue to play a significant role in the management of minor ailments in space, thereby warranting attention to their fabrication under microgravity.

### 5.2 On-demand fabrication of medicines

Following the synthesis of raw ingredients, active pharmaceutical ingredients and excipients must be mixed and fabricated into a dosage form suitable for administration into patients. Three-dimensional printing (3DP), also known as additive manufacturing, is a technology that has been explored for point-of-care fabrication of personalized medicines (Seoane-Viaño et al., 2021c). Through the In-Space Manufacturing project (NASA, 2019b), NASA has already begun including additive manufacturing technologies in missions that will enable on-demand manufacturing and repair capabilities both in-transit and on-surface for logistics and maintenance (Clinton, 2017; NASA, n.d.-b; Werkheiser, 2015). Therefore, given ongoing research on the pharmaceutical applications of 3DP on Earth, it is conceivable that 3DP may eventually be adopted for manufacturing medicines in space. Among the benefits of harnessing this terrestrial technology for space manufacturing, the cost savings derived from the reduction of payload is notable, as well as reducing dependency on spare parts and components (NASA, 2019b).

The first 3D printer to be sent to space in 2014 was a fused deposition modelling (FDM) 3D printer used to create spare parts (NASA, 2019c). FDM is a material extrusion-based technology that heats and extrudes a polymer filament through a nozzle to create a 3D object layer by layer. This technology has been extensively investigated for the fabrication of 3D printed medicines and medical devices on Earth (Cheng et al., 2020; Goyanes et al., 2014). Previous experience in 3DP aboard the ISS have shown that microgravity has no significant effects on the process, paving the way for the use of 3DP in future long-term missions (Prater et al., 2019). In 2017, custom mallet finger splint models were designed and 3D printed in the ISS for hand injuries in astronauts, being the first medical supplies fabricated in orbit (Saunders, 2017). In recent years, smaller, lighter and more efficient 3D printers have reached the market, and some of them have already been adapted or are planned to be adapted to comply with GMP requirements for the production of pharmaceutical products (FabRx, 2020.; Merck, n.d.; DiHeSys, n.d.).

Other material extrusion-based technologies, such as semi-solid extrusion (SSE), and direct powder extrusion (DPE), are promising due to their operating simplicity. SSE is particularly suited for bioprinting purposes as operating temperatures are kept low (Seoane-Viaño et al., 2021a). By using a suitable software, 3D printed tablets (also known as printlets) with the exact dosage tailored to the crewmember's requirements could be printed in a matter of minutes (Elbadawi et al., 2020; Elbadawi et al., 2021; Muñiz Castro et al., 2021; Ong et al., 2022). In the future, it might be possible to exploit the natural resources found on other planets to formulate medications. For example, common excipients used in tableting, such as silica, magnesium silicate and calcium phosphate are found in abundance on the lunar surface and could be leveraged for the preparation of pharmaceutical formulations. This could then lead to an autonomous on-site pharmaceutical manufacturing system. Another possible approach to achieve autonomous manufacturing is by developing a technology that allows the recycling of materials in a similar way to the In-Space Refabricator (NASA, 2018), a hybrid 3D printer that can recycle plastic polymers numerous times to create new items, or the Plastic Recycler (3dnatives, 2019), a manufacturing facility capable of processing polyethylene raw materials into usable 3D printing filaments.

**Fig. 11:**
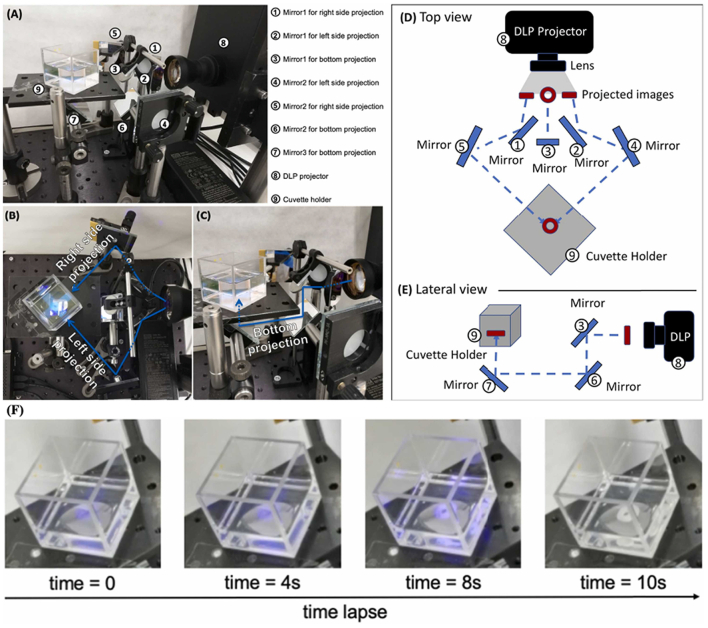
(A-C) Photographs of volumetric printer used to produce printlets loaded with paracetamol (legends 1-9 included on the left). (D-E) Schematic of the volumetric printer system. (F) Sequential images of the printing process. Reproduced with permission from (Rodríguez-Pombo et al., 2022).

Another type of 3D printing that might be suitable for extra-terrestrial application is vat photopolymerization, owing to their speed and precision (Xu et al., 2021a). These technologies (e.g. stereolithography and digital light processing) utilize light to induce the selective solidification of a liquid resin by photopolymerization. Common vat photopolymerization printers use a single wavelength of light to photopolymerize resin in the vat layer-by-layer based on a pre-defined pattern. The light from a smartphone has also been shown to be suitable in serving as the centerpiece for compact 3D printers (3dnatives, 2017; Li et al., 2021; Xu et al., 2021c). Advancements in these light-induced additive manufacturing techniques led to the creation of volumetric 3D printers, which can achieve higher resolutions and complex geometries in a matter of seconds (de Beer et al., 2019; Loterie et al., 2020; Regehly et al., 2020; Rodríguez-Pombo et al., 2022). Rather than using a layer-by-layer approach, volumetric 3D printing projects a light pattern into a vat of liquid resin repeatedly from all angles, creating the entire object simultaneously (Fig. 11).

**Fig. 12:**
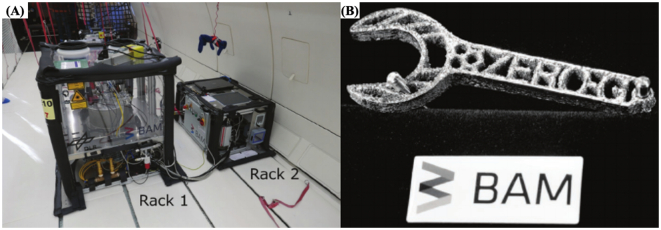
(A) Photo of the setup of the LBM 3D printer mounted in an Airbus A310 ZERO-G. (B) Top view of wrenches manufactured in microgravity. The base plate has a size of 106.5 × 85.5 mm^2^. Reproduced with permission from (Zocca et al., 2019).

Powder bed fusion technologies, such as selective laser sintering (SLS) could also be explored for deployment in space. Recently, a laser beam melting (LBM) 3D printer was tested in zero gravity conditions using metal powders as feedstock materials during a parabolic flight (Fig. 12) (Zocca et al., 2019). They assessed one of the biggest problems with printing in microgravity conditions, which is encountered when the feedstock materials are in the form of powders. In another study, a system capable of printing objects using polystyrene powder as feedstock during a parabolic flight was developed (D’Angelo et al., 2021). The advancement of technologies that allow the use of powdered materials in microgravity may become relevant on sand-covered planets such as Mars, since it will allow the use of regolith (Walton et al., 2007) (the main *in situ* resource) for the manufacture of components, spare parts and tools. In addition, progress made with these technologies could be extrapolated to the fabrication of medicines, since in most cases, raw drugs and excipients are supplied in powder form.

Beyond ad hoc medicinal production, 3D printing can also be used in space to fabricate drug delivery devices that gradually release drug(s) over a specified period of time, or even deliver its drug load to a specific location in the body. For example, gastric retentive devices could easily be manufactured with the help of 3D printing, as in the case of dosage forms to treat malaria where the devices were formed by melt molding the thermoplastic polymers in a mold with a specific geometry created by 3D printing (Bellinger et al., 2016).

Apart from dosage forms, 3D printing can also be used for the fabrication of medical devices that can be drug-loaded. For example, if a crewmember suffers an injury, patches or bandages loaded with drugs that aid in wound healing or possess antimicrobial activity could be printed, improving on-site management of wounds and burns (Albanna et al., 2019; Andriotis et al., 2020). More complex structures, such as bladder devices (Xu et al., 2021b), suppositories (Seoane-Viaño et al., 2020; Seoane-Viaño et al., 2021b) and microneedle patches (Pere et al., 2018; Wu et al., 2020) can also be created using this technology. 3D printing can also be used for bioprinting (Murphy and Atala, 2014). It is projected that continued advancements in bioprinting will soon see researchers able to create complex fully functional cell-laden structures such as skin and ears (Pourchet et al., 2017), corneas (Isaacson et al., 2018) and even organs (Lee et al., 2019) and functional living structures (Duraj-Thatte et al., 2021). These would be useful in accidents and emergency surgeries where an organ transplant or skin graft is required.

Being versatile, 3D printing offers numerous other utilities to enhance the safety and welfare of crew members. Wearable electronic devices capable of monitoring crew members’ health status (including their drug serum concentrations) continuously can be fabricated with 3DP (Ghosh et al., 2018; Hwang et al., 2018; Zhu et al., 2018). Food can also be 3D printed, which can help improve the crew members’ psychological well-being (Karyappa and Hashimoto, 2019; Yang et al., 2018). Functional ingredients, such as probiotics, vitamins and even drugs, may also be incorporated, creating personalized foods that can be tailored according to individual crew member’s nutritional and medical needs and preferences (Liu et al., 2020; Seoane-Viaño et al., 2021a).

By synthesizing the drug molecule with techniques such as Chemputing or synthetic biology, and then including the drug in a suitable pharmaceutical form by 3D printing for administration, a drug manufacturing system almost independent of Earth resources could be achieved. These could be executed on-demand during space missions, resolving the issue of expiration dates and instability of pharmaceuticals stored on board. Admittedly, these presently independent technologies would need to be fully integrated into an automatic or semi-automatic platform to account for the limited number of personnel available onboard to operate them. Further advancements in on-site synthesis of small molecules and biological compounds, along with the versatility of 3D and 4D printing, will lead to the development of smart devices and dosage forms capable of delivering drugs to treat crew members during spaceflights. The potential of these technologies is not limited only to human applications, since they can also be exploited in preclinical studies involving animals, which are very common in the ISS (Brinckmann and Schiller, 2002; Ronca et al., 2019). For example, 3D printed dosage forms adapted in size and dose to small animals, such as mice, may be manufactured and administered on board the ISS. This would facilitate the evaluation of drug pharmacokinetics and pharmacodynamics under microgravity and high radiation conditions, or even to test new drug molecules synthetized on board (Awad et al., 2019; Seoane-Viaño et al., 2020).

Admittedly, the idea of creating an automated drug manufacturing platform capable of functioning in microgravity is still in its infancy. Numerous issues remain, such as downsizing equipment, replacing organic solvents needed in some processes with green alternatives, and developing a system for waste processing and recycling. In addition, to operate in microgravity conditions it will need extensive development and testing during space travel or on the ISS. Moreover, it will require user-friendly software that includes quality control tests to ensure the safety of the medicines produced.

## 6 Ensuring efficacy and safety: on-site drug analysis

**Fig. 13:**
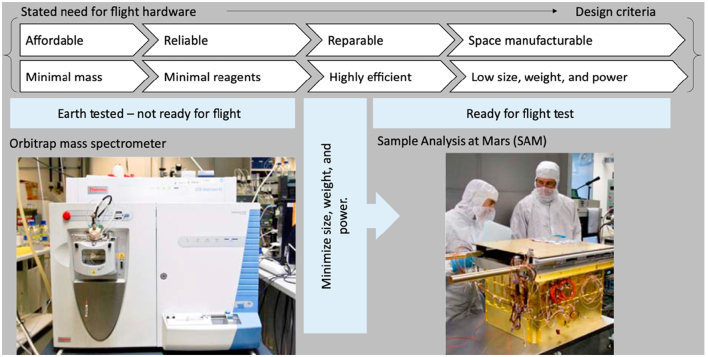
Diagram describing the design criteria for equipment in space. Reproduced with permission from (Snyder et al., 2019).

Following the preparation of medicines, it is essential to assure the quality of the dosage forms and drugs obtained to guarantee patient safety and clinical efficacy. Conventionally, quality control (QC) tests used in large-scale manufacturing processes on Earth aim to establish shelf-life and identify by-products by accelerating abiotic degradation processes, such as hydrolysis, oxidation and photolysis (EMA, 2003). Analysis typically involves multiple techniques including gas chromatography – mass spectrometry (GC/MS), infra-red spectroscopy (IR), nuclear magnetic resonance spectroscopy (NMR), and elemental analysis techniques (Sohail Arshad et al., 2021). These help to identify the presence of any impurities, such as heavy metals and leachable, and degradation products due to drug instability. Determining the presence of benign or toxic degradation products is particularly relevant for pharmaceuticals in space, given that elevated radiation in space may accelerate the degradation of drugs (Du et al., 2011). Failure in doing so would result in reduced efficacy or might cause adverse side effects or even death if toxic (Song and Chen, 2001). QC tests also aim to ensure the performance of medicines meets their marketed claims. This include ensuring consistent drug content and release behavior, which involves in-vitro dissolution tests. However, these QC processes are destructive, labor intensive, and require heavy machinery. Effort has been made to adapt machinery on Earth to the requirements for spaceflight (Fig. 13). However, the destructive nature of these processes prevents them from fitting into a just-in-time model where medicines are produced as required and cannot be destroyed for analysis.

Therefore, a non-destructive and portable means of quality control is necessary to support on-demand medicine production in space. Technological advances made in recent years have made Raman spectroscopy faster and more reliable. Advances in component miniaturization allowed the development of lightweight, portable and handheld devices well-suited for pharmaceutical applications from testing of raw materials to quality control of finished products (Paudel et al., 2015). As such, handheld Raman devices could be easily carried on board to analyse unit doses immediately prior to use during long-term space missions. This has been demonstrated in a study conducted on Earth, in which Raman spectroscopy was used to determine the degree of degradation of drugs in a non-destructive manner and without sample preparation in 10 min or less (Shende et al., 2014). The device evaluates the active ingredient content by comparing the Raman spectrum it measures with stored spectra of effective drugs and those that have degraded.

Several studies have demonstrated the use of non-destructive analytical techniques for real time drug quantification (Edinger et al., 2017; Edinger et al., 2018). Notably, Raman and NIR spectroscopy have been applied to perform QC measures on 3D printed medicines (Trenfield et al., 2018; Trenfield et al., 2020). It is expected that non-destructive analytical techniques coupled with Artificial Intelligence (AI)-powered analysis can correlate formulation architecture design with important parameters, such as dissolution behaviour and drug release kinetics. Machine learning models could predict excipient combinations to achieve specific dissolution profiles or enhance bioavailability (Gao et al., 2021; Windbergs et al., 2010). These technological advances are increasingly bringing the possibility of manufacturing safe and effective medicines *in situ* on board quickly and with minimal resources.

## 7 Future perspectives

The 21^st^ century space race will bring many far-reaching advancements in science and technology. Notably, technologies developed for extra-terrestrial applications could also be deployed in hard-to-reach areas on Earth. For instance, isolated villages in rural regions and military field hospitals could benefit from technologies that enable the on-site production of medicines and medical devices using fewer resources or locally sourced materials, thereby becoming self-sustainable (Seoane-Viaño et al., 2021c). Examples of technologies that were initially developed for use in space but later also used on Earth include artificial limbs, insulin pumps, cochlear implants, infrared ear thermometers and invisible braces (NASA, n.d.-a). Therefore, investments in innovative medical technologies could reap dual benefits, both in supporting mankind’s ambitions in deep space and in improving healthcare provision on Earth (Awad et al., 2021).

The vibrant research community involved in advancing point-of-care medical manufacturing and analytical technologies provide optimism that the envisioned model of just-in-time medications in space might soon become a reality. However, technological advancements must be accompanied by the introduction of new regulatory frameworks to safeguard patient safety and public confidence in these emerging technologies (Arden et al., 2021). This is particularly important in this case as existing regulations, manufacturing processes and quality control procedures used on Earth cannot be directly applied in long-term space missions. Therefore, as alluded to earlier, it might be pertinent to establish an international medicine agency dedicated to the safety and efficacy and medicines in space – the “Space Medicines Agency” (SMA).

Apart from facilitating clinical trials in space as aforementioned, the SMA should control the assessment and approval of new manufacturing or analytical devices. This assessment may be adapted from Good Manufacturing Practice (GMP) requirements that are currently enforced on Earth (Hole et al., 2021). In view of emerging technologies, this might warrant additional work since many of these innovations have yet to meet conventional GMP requirements. For instance, except for a few 3D printers that have been validated against GMP regulations or are currently under development (FabRx, 2020; Health, n.d.; Industries, n.d.; Medical, n.d.; DiHeSys, n.d.), the 3D printers used in academic research papers do not meet GMP requirements. Given the rapid pace at which these technologies are being developed, it might be appropriate to begin dialogue on how these regulatory requirements need to be adapted for application in space. Other functions of the SMA may include accrediting training of staff in space, such as pharmacists for tasks related to medication management and provision of medication information (Sawyers et al., 2022).

**Fig. 14:**
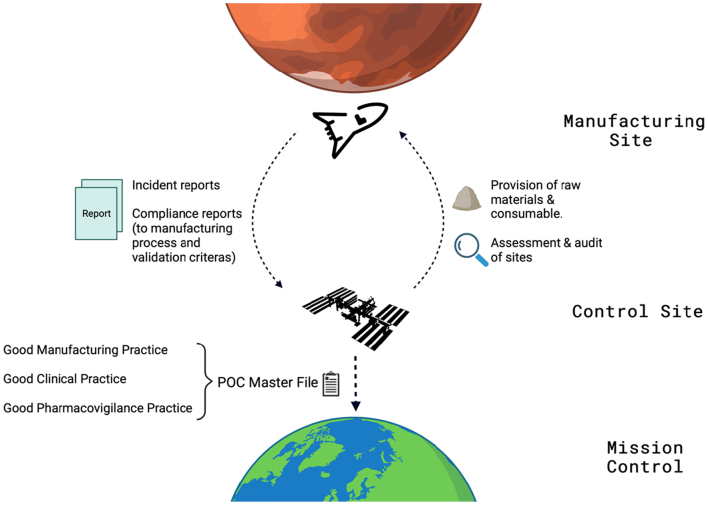
Schematic illustrating a proposed regulatory framework. Created with BioRender.com.

For the SMA to effectively perform these duties, proximity to the site of manufacture and dispensing is critical. A physical site in space might therefore be necessary for the SMA to perform its functions. Inspiration can be drawn from the Control Site concept proposed in the Medicines & Healthcare products Regulatory Agency (MHRA) Consultation on Point of Care manufacturing. Under this system, the Control Site will “be responsible for overseeing all aspects of the [Point of Care] manufacturing system including the addition of new manufacturing sites and control of each manufacturing location and their activities” (MHRA, 2021). As shown in Fig. 14, the Control Site would oversee the provision of raw materials and would receive incident and compliance reports from the Manufacturing Site. In space, the Control Site may be a spaceship that is located centrally amongst other spacecrafts carrying crew members on active missions. The Control Site will periodically feedback a “POC Master file”, containing regulatory and inspection documents, to the SMA Mission control center on Earth.

The aforementioned system is only one plausible model of safeguarding the quality of medicines manufactured on board spacecrafts. With the imminent advent of space tourism, establishing a robust system of medicine provision will be crucial for assuring the health and safety of customers during their residence in space. Therefore, active discussion on a system of medicines regulation should now begin to facilitate the smooth adoption of emerging medical technologies in space.

## 8 Conclusions

As a new era of space exploration begins, investments towards deep space exploration and extra-terrestrial colonization should not be limited to advancements in aerospace engineering. Maintaining the health of crew members will be crucial for mission success, and to achieve this, it will be necessary to develop technologies that enable and support the on-demand production of medicines in space. With deep space exploration, resupply missions from Earth may not be feasible. The limited shelf life of drugs, potentially aggravated by accelerated degradation due to exposure to space radiation, makes it almost impossible to rely on medicines stored on board. Moreover, given the altered pharmacokinetics of drugs during space travel, it is necessary to deploy technologies that facilitate the fabrication and administration of drugs at a precise dose tailored to an individual patient's needs. Beyond technological advances, a robust system of medicines regulation must be established to ensure the quality of medicines manufactured on board spacecrafts. With continued enthusiasm and active dialogue on foreseeable challenges, it will not be long before humankind takes its next giant leap.

## CRediT authorship contribution statement

**Iria Seoane-Viaño:** Conceptualization, Investigation, Writing – original draft, Writing – review & editing. **Jun Jie Ong:** Conceptualization, Investigation, Writing – original draft, Writing – review & editing. **Abdul W. Basit:** Supervision, Writing – review & editing. **Alvaro Goyanes:** Conceptualization, Investigation, Supervision, Writing – review & editing.

## Declaration of Competing Interest

The authors declare that they have no known competing financial interests or personal relationships that could have appeared to influence the work reported in this paper.
